# Opportunistic prediction of osteoporosis in patients with degenerative lumbar diseases: a simplified T12 vertebral bone quality approach

**DOI:** 10.1186/s13018-024-04782-0

**Published:** 2024-05-15

**Authors:** Congyang Xue, Xiaopei Lu, Guangda Sun, Nan Wang, Ganshen He, Wenqiang Xu, Zhipeng Xi, Lin Xie

**Affiliations:** 1 Department of Orthopedic, Affiliated Hospital of Integrated Traditional Chinese and Western Medicine, Nanjing University of Chinese Medicine, 100th. Shizi Street, Nanjing, Jiangsu Province 210028 P.R. China; 2 Nanjing University of Chinese Medicine, Nanjing, Jiangsu 210023 China

**Keywords:** Osteoporosis, Vertebral bone quality, Hounsfield units, Thoracic vertebra, MRI

## Abstract

**Background:**

Osteoporosis is one of the risk factors for screw loosening after lumbar fusion. However, the probability of preoperative osteoporosis screening in patients with lumbar degenerative disease is low. Therefore, the aim of this study was to investigate whether a simplified vertebral bone quality (VBQ) score based on T12 T1-MRI could opportunistically predict osteoporosis in patients with degenerative lumbar spine diseases.

**Methods:**

We retrospectively analyzed cases treated for lumbar degenerative diseases at a single institution between August 2021 and June 2022. The patients were divided into three groups by the lowest T-score: osteoporosis group, osteopenia group, and normal bone mineral density (BMD) group. The signal intensity based on the T12 vertebral body divided by the signal intensity of the cerebrospinal fluid was calculated to obtain the simplified VBQ score, as well as the CT-based T12HU value and the traditional L1-4VBQ score. Various statistical analyses were used to compare VBQ, HU and DEXA, and the optimal T12VBQ threshold for predicting osteoporosis was obtained by plotting the receiver operating curve (ROC) analysis.

**Results:**

Total of 166 patients were included in this study. There was a statistically significant difference in T12VBQ scores between the three groups (*p* < 0.001). Pearson correlation showed that there was a moderate correlation between T12VBQ and T-score (*r*=-0.406, *p* < 0.001). The AUC value of T12VBQ, which distinguishes between normal and low BMD, was 0.756, and the optimal diagnostic threshold was 2.94. The AUC value of T12VBQ, which distinguishes osteoporosis from non-osteoporosis, was 0.634, and the optimal diagnostic threshold was 3.18.

**Conclusion:**

T12VBQ can be used as an effective opportunistic screening method for osteoporosis in patients with lumbar degenerative diseases. It can be used as a supplement to the evaluation of DEXA and preoperative evaluation.

**Trial registration:**

retrospectively registered number:1502-009-644; retrospectively registered number date:27 oct 2022.

## Introduction

Degenerative diseases of the lumbar spine are characterized by low back and leg pain as the main clinical symptom [1]. Its prevalence gradually increases over time; without timely intervention, symptoms gradually worsen. The main common lumbar degenerative diseases include discogenic low back pain, lumbar disc herniation [2], lumbar spinal stenosis [3, 4] and lumbar spondylolisthesis [5]. It is one of the major causes of disability in elderly patients. Currently, most of the severe degenerative lumbar spine diseases are treated surgically. Approximately 600,000 lumbar spinal stenosis surgeries are performed annually in the United States [4]. Some patients are in need of lumbar spinal stenosis fusion surgery [6]. Osteoporosis was found to be a risk factor for cage settlement after lumbar fusion [7] and one of the main risk factors for pedicle screw loosening [8]. Therefore, early identification of patients with bone abnormalities facilitates the optimization of the choice of surgical approach and postoperative care, and reduces the incidence of associated complications.

However, the probability of screening for osteoporosis in patients with degenerative lumbar spine disease is low. A previous survey of clinicians found that only 47% of clinicians screened patients for osteoporosis before surgery [9]. Although, DEXA test is the gold standard for diagnosing osteoporosis [10]. But due to many factors [11] (such as vertebral compression fractures, degenerative joint disease, scoliosis and vascular calcification, etc.), it does not detect osteoporosis effectively. In the clinic, osteoporosis is found in patients mostly after the first fracture. One study [12] found that more than 50% of patients with osteoporotic fractures had T-score > -2.5. In contrast, q-CT, although better able to assess a patient’s bone density, is expensive and has high radiation and low clinical utilisation. As a result, this has prompted researchers to look for other ways to predict the bone density of vertebrae.

The investigators developed methods to opportunistically screen patients for osteoporosis based on imaging data from their preoperative evaluation [13, 14]. Currently, T12HU values based on CT measurements are effective in assessing patient bone quality [15]. MRI-based vertebral bone quality scores for the prediction of osteoporosis have been proposed with the ability to assess the quality of bone trabeculae and the degree of fat infiltration. The study found a significant correlation between VBQ and BMD [16]. Many researchers have used VBQ as an indicator to assess cage subsidence and recurrence after lumbar fusion surgery [7, 17]. However, deformities, fractures, hemangiomas and local infections of the lumbar spine may affect the L1-4VBQ score. Therefore, we were curious if MRI data of the T12 could be used to simplify the VBQ score, which was not mentioned in previous studies.

The aim of this study was to investigate whether a simplified VBQ score based on T12 T1-MRI could opportunistically predict osteoporosis in patients with degenerative lumbar spine disease; To evaluate the correlation between DEXA T values and T12HU and T12VBQ scores; and to determine the T12VBQ threshold for opportunistic screening for osteoporosis.

## Materials and methods

### Patients’ population

Among the patients who attended and were admitted to the Department of Orthopaedics of Affiliated Hospital of Integrated Traditional Chinese and Western Medicine, Nanjing University of Chinese Medicine between 1 August 2021 and 30 June 2022, we reviewed the relevant medical data of 535 patients with the presence of lumbar degenerative diseases. Inclusion criteria: (1) age ≥ 18 years; (2) MRI, CT and DXA performed at our hospital. Exclusion criteria: (1) patients with incomplete imaging information (e.g., CT images not scanned to T12; no T1-weighted MRI; patients with fracture present in T12/L1-L4, etc.) or bone mineral density information; (2) patients suffering from diseases affecting bone metabolism (e.g., hyperthyroidism, etc.) or taking medications affecting bone metabolism (e.g., use of glucocorticosteroids, etc.); (3) lack of necessary data, etc. A final total of 166 patients were included. The design of the study was approved by the Ethics Review Committee of our hospital (IRB No. 1502-009-644). The Institutional Review Board waived the need for written informed consent from participants.

### DEXA data acquisition

All patients were tested at the lumbar spine (L1-4) and hip by dual-energy X-ray absorptiometry to obtain T values at the lumbar spine and hip. The patients were finally assessed and analysed with the lowest T value between the two. According to the World Health Organisation criteria [10], osteoporosis was defined as a T-score ≤ -2.5, osteopenia was defined as -2.5 < T-score ≤ -1.0, and normal BMD as a T-score > -1.0. All the patients were carried out into osteoporosis group, osteopenia group and normal BMD group.

### Measurement of vertebral HU

First, the sagittal position of the CT image was selected to determine the measurement position; then a region of interest (ROI) was drawn on the axial position of the corresponding vertebral bone trabeculae to make it as large as possible, but excluding the vertebral cortical bone, surrounding venous plexus, and trophoblastic foramen. Finally, the HU values of the near upper endplate, the middle, and the near lower endplate were measured separately, and then averaged [14].

### VBQ measurement of T12 and L1-4

**Fig. 1:**
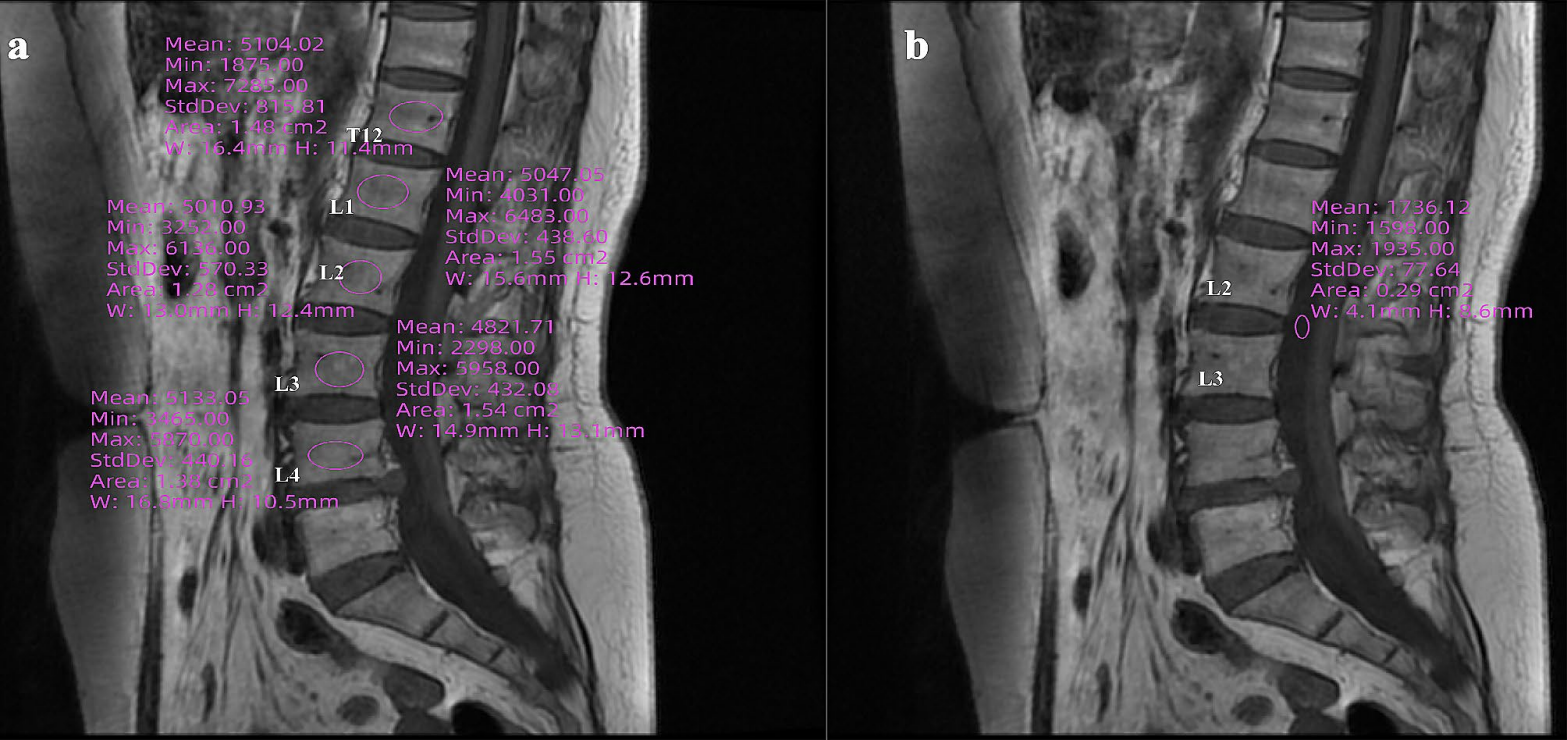
Schematic diagram of VBQ score measurement. Figure 1**a** shows the process of measuring SI values of T12-L4 vertebrae; Fig. 1**b** shows the process of measuring SI values of cerebrospinal fluid at the L3 level

Measurement of L1-4 VBQ according to Ehresman and other researchers [13, 18]. The first step was to select the sagittal position of the T1-weighted MR image of the lumbar spine; the second step was to draw a circular area on the trabeculae of the L1-4 (excluding cortical bone, focal lesions (metastatic lesions or hemangiomas) and the posterior venous plexus, respectively) in order to obtain the signal intensities (SI) of the four vertebral bodies and to take the average of the four; and in the third step, to create a ROI at the cerebrospinal fluid in the position at the level of the L3 level, and to measure the SI of the cerebrospinal fluid (see Fig. 1). Finally, the VBQ scores for L1-4 were calculated according to the following formula:

L1-4VBQ = SI_L1−4_/CSF_L3_

To assess whether the method could be simplified, we applied a similar method to measure the SI value of the T12 vertebrae; the VBQ score for T12 was calculated according to the following formula:

T12 VBQ = SI_T12_/CSF_L3_

All measurements were performed by a physician who was unaware of the DXA results. Another author randomized 20 patients for comparison of measurements.

### Statistical analysis

All data were stored in Microsoft Excel. Data were analyzed using GraphPad prism 9 (GraphPad software, LLC, California, USA). Continuous variables (e.g., age, BMI, etc.) were expressed by means and standard deviations, and comparisons between groups were made by t-test or one-way ANOVA; categorical variables (e.g., gender, comorbidities, etc.) were expressed using percentages and analyzed by chi-square test. Pearson correlation was used to analyze the correlation between T12HU, T12VBQ and T-Score. ROC curves were plotted to assess the predictive value of VBQ scores for bone loss and osteoporosis. p-value < 0.05, statistically significant.

## Results

### Patient characteristics

A total of 166 patients were included in this study and their basic information data were stored in Table 1. Based on the lowest T-value of the patients, all the patients were categorized into three groups: osteoporosis group, osteopenia group and normal BMD group. Statistical analysis revealed statistically significant differences between the three groups of patients in terms of age, gender, BMD, T-values (lumbar/hip), and T12/L1-4 VBQ values and T12 HU. There was no statistically significant difference between the underlying disease (hypertension/diabetes), lifestyle (smoking/alcohol abuse) and BMI in the three groups, although there were differences.

**Table 1:** Patient demographics

	Osteoporosis(*n* = 32)	Osteopenia(*n* = 69)	Normal(*n* = 65)	*P* value
*Characteristics*				
Age	70.3 ± 10.6	67.7 ± 9.5	59.7 ± 13.6	<0.001
*gender*				0.002
Male	5(15.6%)	22(31.9%)	33(50.8%)	
Female	27(84.4%)	47(68.1%)	32(49.2%)	
BMI	23.3 ± 2.5	23.9 ± 3.3	24.7 ± 3.3	0.098
diabetes	3(9.3%)	9(13.0%)	11(16.9%)	0.357
hypertension	12(37.5%)	24(34.8%)	20(30.8%)	0.263
Cigarette	2(6.2%)	5(7.2%)	10(15.4%)	0.415
Alcoholism	1(3.1%)	2(2.9%)	8(12.3%)	0.453
*DEXA T-score*				
Lumbar T-score	-2.87 ± 0.46	-1.55 ± 0.56	0.29 ± 0.96	<0.001
Hip T-score	-1.51 ± 0.93	-0.71 ± 0.86	0.71 ± 1.12	<0.001
Lowest T-score	-2.93 ± 0.35	-1.71 ± 0.39	0.64 ± 1.07	<0.001
*DEXA BMD*				
Lumbar BMD	0.77 ± 0.06	0.95 ± 0.08	1.22 ± 0.14	<0.001
Hip BMD	0.75 ± 0.11	0.85 ± 0.11	1.03 ± 0.14	<0.001
L1-4 VBQ	3.56 ± 0.73	3.52 ± 0.69	2.89 ± 0.74	<0.001
T12 VBQ	3.61 ± 0.72	3.59 ± 0.72	2.91 ± 0.75	<0.001

BMI body mass index, DEXA dual-energy X-ray absorptiometry, VBQ vertebral bone quality, HU Hounsfield units, L lumbar, T thoracic

### Pearson correlation analysis

**Table 2:** Correlation between T values and T12HU, T12VBQ and L1-4VBQ

Pairs	*R* value	*P* value
T12 HU and T-score	0.531	<0.001
T12 VBQ and T-score	-0.406	<0.001
T12 HU and T12 VBQ	-0.416	<0.001
T12 VBQ and L1-4 VBQ	0.907	<0.001

Using Pearson correlation analysis, a very strong correlation was found between T12VBQ and L1-4VBQ, as well as a strong positive correlation between T12HU and T values; There was a strong negative correlation between T12VBQ values and T12HU and T values, indicating that the higher the VBQ score, the smaller the vertebral HU and T values; the correlation between the indicators in each group was statistically significant (*p* < 0.001). (see Table 2)

### ROC curve analysis

**Fig. 2:**
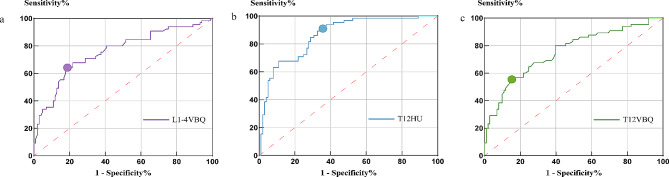
Receiver operating characteristic curve (ROC) for L1-4VBQ and T12HU/VBQ. Figure 2**a** is the ROC for L1-4VBQ shows that L1-4VBQ had an AUC value of 0.757, with an optimal diagnostic threshold of 2.98; Fig. 2**b** is the ROC for T12HU shows that T12HU had an AUC value of 0.863, with an optimal diagnostic threshold of 102.1; Fig. 2**c** is the ROC for T12VBQ shows that T12VBQ had an AUC value of 0.756, with an optimal diagnostic threshold of 2.94

ROC curve analysis was used to analyze the diagnostic accuracy of L1-4VBQ and T12HU/VBQ in normal and abnormal BMD (see Fig. 2). Among them, L1-4VBQ had an AUC value of 0.757, with an optimal diagnostic threshold of 2.98; T12HU had an AUC value of 0.863, with an optimal diagnostic threshold of 102.1; and T12VBQ had an AUC value of 0.756, with an optimal diagnostic threshold of 2.94. The AUC values for T12HU were significantly greater than those for L1-4ABQ and T12VBQ scores, whereas the AUC values for L1-4VBQ scores and T12VBQ scores were almost identical.

**Fig. 3:**
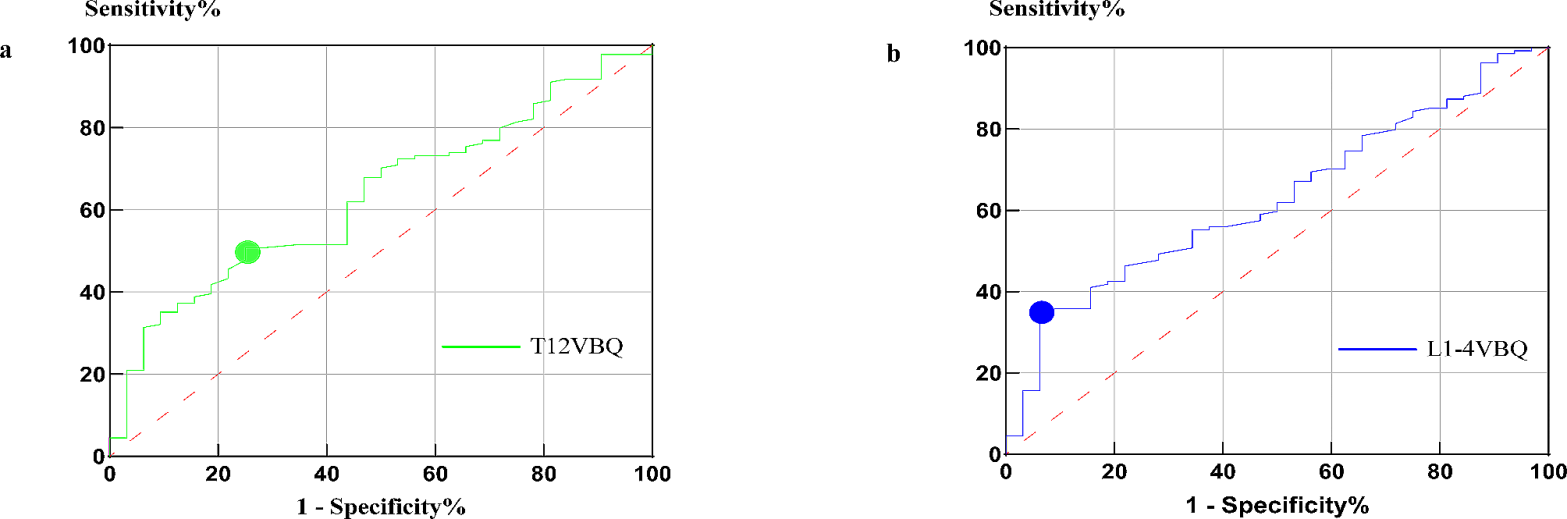
ROC analysis of L1-4VBQ and T12VBQ in osteoporotic versus non-osteoporotic patients. Figure 3**a** shows the ROC of L1-4VBQ in subjects with osteoporosis versus non-osteoporotic patients; Fig. 3**b** shows the ROC of T12 VBQ in subjects with osteoporosis versus non-osteoporotic patients

The optimal thresholds for the T12 and L1-4 VBQ in distinguishing osteoporosis from non-osteoporosis were determined by ROC curve analysis (Fig. 3). The AUC value of the T12 VBQ was 0.634, and the optimal diagnostic threshold was 3.18; the threshold for scoring the T12 VBQ with high sensitivity (90%) was 4.43, and the threshold for scoring the T12 VBQ with high specificity (90%) was 2.90. The AUC value of the L1-4 VBQ was 0.629, and the optimal diagnostic threshold was 2.85.

**Table 3:** Diagnostic value of VBQ thresholds in differentiating between normal and low BMD and in differentiating between osteoporosis and non-osteoporosis in comparison with previous studies

variable	Distinguishing normal from low bone mineral density			Distinguishing osteoporosis from non-osteoporosis		
	Current study (T12VBQ)	Current study (L1-4VBQ)	L1-4VBQ	Current study (T12VBQ)	Current study (L1-4VBQ)	L1-4VBQ
Cutoff VBQ	2.94	2.98	3.06	3.18	2.85	2.7
Sensitivity (95% CI), %	56.9(44.8–68.2)	64.6(52.5–75.1)	63.6(-)	50.8(42.38–59.07)	35.1(27.5–43.5)	83.3(-)
Specificity (95% CI), %	85.2(76.9–90.8)	82.1(73.6–88.4)	87.0(-)	75.0(57.9–86.8)	93.8(79.9–98.9)	44.3(-)
PPV %	75.4	78.3	94.2	26.7	25.6	25.3
NPV %	71.2	70.0	41.7	89.5	95.9	92.2

In the present study, we found that the optimal thresholds of T12VBQ and L1-4VBQ in differentiating between normal and low BMD were almost the same as, and slightly lower than, those of the study by Pu et al [19]. The negative predictive value of T12VBQ obtained in this study was significantly better than that of Pu et al.; The optimal threshold of T12VBQ in distinguishing osteoporosis from non-osteoporosis was significantly higher than that of L1-4VBQ. The specificity of the VBQ obtained in the present study was significantly better than that of the study by Ozmen et al [20]. The sensitivity was poorer, and both studies were similar in terms of negative and positive prediction rates. (see Table 3)

## Discussion

To the best of our knowledge, this is the first study to examine whether the T12 VBQ can be used as an opportunistic screen for osteoporosis. The results of this study showed that the T12 VBQ had an accuracy of 0.634 for the diagnosis of osteoporosis versus non-osteoporosis, and 0.756 for the diagnosis of normal versus low BMD, which shows that it is able to opportunistically screen patients for the presence of osteoporosis or low BMD.

Despite the increasing prevalence of osteoporosis, only 27% of patients eligible for osteoporosis screening had DEXA tests performed [21]. Female patients are more prone to osteoporosis than male patients, so the US Preventative Services Task Force only recommends DEXA screening for women, which may result in lower screening rates for men [22]. Men have a higher prevalence of lumbar degenerative diseases compared to women [23].Therefore, the preoperative evaluation of many patients requiring surgery lacks DEXA.

For opportunistic screening for osteoporosis, MRI offers advantages that CT does not. The gold standard for the diagnosis of osteoporosis is still the DEXA test, but the results are often high and cause false negatives due to the effects of calcification of the vessel wall, synovial hyperplasia, and degenerative bone spurs [24]. Although Q-CT can be used for early diagnosis of osteoporosis, its high cost of equipment, complexity of post-processing analyses and high radiation exposure limit its use in clinical practice. At present, numerous studies have found that CT-based trabecular HU values and MRI-based VBQ can be used for opportunistic screening for osteoporosis [13, 24]. Our group found that the accuracy of T12HU for the diagnosis of low BMD was 0.863, which was significantly higher than the VBQ score. However, screening for osteoporosis based on CT HU also has a number of limitations, such as the effects of radiation and equipment-to-equipment variation. However, VBQ values based on T1WI have certain advantages. Since MRI is able to directly assess the structure of nerves and intervertebral discs; therefore, it is more accessible to clinicians in degenerative disc disease. Meanwhile, MRI is radiation-free, protects patients from secondary injuries, and is less affected by differences in equipment.

T12VBQ is simpler and more practical than L1-4VBQ, while the combination of T12HU and T12VBQ facilitates the accuracy of opportunistic screening for osteoporosis. When a fracture occurs in the lumbar spine, the accuracy of L1-4VBQ will be affected; if T12VBQ is utilised, it will not be affected by the lumbar spine fracture. Meanwhile, for surgeries involving the T12, the T12VBQ more accurately reflects the T12 bone quality. Using Pearson’s correlation analysis, it was found that there was a negative correlation between T12VBQ and T-value, i.e., the higher the T12VBQ value, the worse the bone quality of the patient. The correlation between T12HU and T-values was greater than the correlation between T12VBQ and T-values, suggesting that HU is superior to VBQ, which is consistent with previous studies [7, 25, 26]. This may be because CT directly assesses the quality of bone trabeculae, whereas VBQ indirectly assesses bone density by measuring the signal intensity of fat. There was also some negative correlation between T12HU and T12VBQ. The correlation between all three may reflect an intrinsic link between the three modalities for assessing BMD. Patients with degenerative lumbar spine disease requiring surgery often have imaging data from lumbar CT and MRI, and it is recommended that HU be assessed in combination with VBQ scores to improve accuracy.

Using ROC curve analysis to assess patients with normal and low bone mineral density found that the threshold and AUC area of the T12VBQ were similar to that of the L1-4VBQ, with a difference of 0.04 and 0.01, respectively. The sensitivity of the T12VBQ threshold was slightly worse than that of the L1-4VBQ, but the specificity was higher than that of the L1-4VBQ. Both positive and negative predictive values ranged from 70 to 79%. It suggests that both have almost the same diagnostic performance for normal and low bone mineral density. The T12VBQ threshold obtained in this study to distinguish between normal and low BMD was smaller than the L1-4VBQ threshold of 3.06 proposed by Pu and other researchers, but the difference between the two was not significant and the negative predictive value was significantly higher than that of Pu’s study [19]. Huang et al. proposed a threshold of 2.93 for the S1VBQ to distinguish between normal and low BMD, which is similar to the results of this study [25]. The threshold of T12VBQ obtained in distinguishing osteoporosis from non-osteoporosis was 3.18, which was significantly higher than the L1-4VBQ threshold (2.85) obtained in this study and the L1-4VBQ threshold (2.7) proposed by Ozmen et al [20]. However, it is similar to the thresholds proposed by researchers such as Pu et al. The specificity of the T12VBQ threshold for distinguishing osteoporotic from no osteoporotic T12VBQ derived from the present study was significantly higher than that of the Ozmen et al. study. When Ozmen et al. adjusted the threshold to 3, the sensitivity was 54%, specificity was 73%, PPV was 31% and NPV was 88%, which is more similar to the results of the present study.

Previous studies have proposed that VBQ and age are significant predictors of osteoporosis [25], which is consistent with the present study. One study found that low BMI is one of the influencing factors of osteoporosis [27]. Although there was a difference in BMI among the three groups of patients in this study, the difference was not statistically significant. This may be due to the higher BMI of patients with degenerative lumbar spine disease included in this study [28]. Clinical studies have found that diabetic patients are more prone to fracture although their BMD is higher than non-diabetic patients [29]. There was no statistically significant difference in the prevalence of underlying diseases (hypertension/diabetes) among the three groups of patients in this study. It is suggested that chronic metabolic diseases such as diabetes and hypertension, although they can influence the development of osteoporosis, cannot be detected in time based on DEXA examination.

The present study also has limitations: firstly, the low rate of osteoporosis included in the study may be due to the fact that this study included patients with lumbar degenerative diseases, which are more likely to have bone encumbrances than the general population, thus affecting the DEXA examination; secondly, the proportion of female included in this study is low, which is due to the fact that the prevalence of male patients with lumbar degenerative diseases is higher; however, it also overcame the disadvantage that the sample of the previous study was mostly female, which made our study more applicable to the Asian population; this study included patients with lumbar degenerative diseases, so there may be a selection bias, and the source of the data should be further expanded in the future.

## Conclusion

Our simplified VBQ score using T12 T1-weight MRI is valuable for evaluating bone quality in patients with lumbar degenerative disease. It is recommended that the VBQ score may be included in imaging department reports to help physicians evaluate whether patients need further osteoporosis screening.

## Data Availability

No datasets were generated or analysed during the current study.
